# Hit-to-lead and lead optimization binding free energy calculations for G protein-coupled receptors

**DOI:** 10.1098/rsfs.2019.0128

**Published:** 2020-10-16

**Authors:** Shunzhou Wan, Andrew Potterton, Fouad S. Husseini, David W. Wright, Alexander Heifetz, Maciej Malawski, Andrea Townsend-Nicholson, Peter V. Coveney

**Affiliations:** 1 Centre for Computational Science, Department of Chemistry, University College London, London WC1H 0AJ, UK; 2 Institute of Structural and Molecular Biology, Research Department of Structural and Molecular Biology, Division of Biosciences, University College London, London WC1E 6BT, UK; 3 Evotec (UK) Ltd, 114 Innovation Drive, Milton Park, Abingdon OX14 4RZ, UK; 4 ACK Cyfronet, AGH University of Science and Technology, Nawojki 11, 30-950, Kraków, Poland; 5 Computational Science Laboratory, Institute for Informatics, Faculty of Science, University of Amsterdam, 1098XH Amsterdam, The Netherlands

**Keywords:** molecular dynamics, free energy, binding affinity prediction, G protein-coupled receptors, adenosine receptors, ensemble simulations

## Abstract

We apply the hit-to-lead ESMACS (enhanced sampling of molecular dynamics with approximation of continuum solvent) and lead-optimization TIES (thermodynamic integration with enhanced sampling) methods to compute the binding free energies of a series of ligands at the A_1_ and A_2A_ adenosine receptors, members of a subclass of the GPCR (G protein-coupled receptor) superfamily. Our predicted binding free energies, calculated using ESMACS, show a good correlation with previously reported experimental values of the ligands studied. Relative binding free energies, calculated using TIES, accurately predict experimentally determined values within a mean absolute error of approximately 1 kcal mol^−1^. Our methodology may be applied widely within the GPCR superfamily and to other small molecule–receptor protein systems.

## 1. Introduction

There is an urgent need for approaches and tools that permit the prediction of rapid, accurate and reliable properties of systems across science as a whole. We have a longstanding interest in the development of *in silico* methodologies able to predict values computationally that agree with and therefore may replace experimental measurements [1–4]. Here, we focus our efforts on a subject of global importance in computational biomedicine: the accurate prediction of protein–small molecule binding affinities. The calculation of accurate binding affinities will provide substantial insight into ligand–receptor interactions for scientists, significantly impact the drug discovery process in industry and expedite the implementation of personalized medicine, making it more commercially attractive and facilitating the development of bespoke, individualized, pharmaceuticals to significantly improve patient prospects and bring about economic savings for healthcare programmes. The prediction of binding free energies is a computationally tractable task that, with iteration, can provide a self-reinforcing loop between experimental data and theoretical calculations.

Binding free energy can be calculated using pathway or endpoint methods. A pathway can be either a physical binding path or an alchemical path. The former is usually defined by a suitable collective variable with which simulation is driven and free energy change is derived. Large conformational space needs to be sampled along the binding path, which usually requires enhanced sampling approaches. Recently a metadynamics protocol has been applied for ligand binding free energies to G protein-coupled receptors (GPCRs) [5,6], generating encouraging results. The approach is valuable to explore the pathways of ligand binding, to find the binding site(s), to predict the binding poses and to estimate binding free energies. However, such simulations require a timescale of microseconds and need to run for days if not weeks on high performance supercomputers. The rapid development of computational power may make it possible for these techniques to deliver actionable predictions within ten years. The metadynamics protocol, however, is unable to satisfy the requirement for pharmaceutical drug development today. Alchemical and endpoint approaches, on the other hand, are being increasingly promoted by pharmaceutical companies collectively [7] as they can be implemented in a rapid, accurate and reliable manner [8].

To accurately and reliably compute these values, it is necessary to appreciate that macromolecular biological systems are capable of adopting various conformations depending on how they are studied by simulation and that results obtained from single trajectory (one-off) simulations—particularly long ones—lack the accuracy and reproducibility needed for convergence with experimentally determined values [9]. This is only now becoming fully understood by many practitioners of molecular simulation. Despite this, single trajectory approaches that use methods such as MMPBSA (molecular mechanics Poisson Boltzmann surface area) [10], WaterMap (WM) [11], and the semi-empirical, linear interaction method (LIE) [12,13] have been used extensively. Although the benefit of using ensembles comprised of multiple simulations, or replicas, has been demonstrated [14], and applied to the calculation of free energies [15] it is only recently that new methods have been introduced which improve sampling and accessibility of the conformational space in molecular dynamics (MD)-produced trajectories [3,16–18]. One of these, ESMACS [2,14,16,17,19] (enhanced sampling of molecular dynamics with approximation of continuum solvent), uses ensembles of multiple and typically relatively short duration simulations to calculate absolute binding free energies with high precision. There is a wealth of evidence in the literature and in unpublished work that, under equilibrium conditions, multiple short MD simulations sample better than a single long MD simulation and provide a meaningful uncertainty of the results [8,9,20–22]. Under general non-equilibrium conditions, ensembles are essential since there is then no meaning to time averaging. Here, we use an ensemble approach for precise sampling of restricted regions of conformational space which are important for the calculation of the properties of interest. Many other properties, such as kinetics and transition rates, require sampling of a much larger conformational space. Long time scale simulations will be needed, usually with accelerated methods such as metadynamics. It should be noted that single long time simulations are inaccurate, as we have explained before (e.g. [22]). Ensembles are required for all MD simulations [20,22], as precise predictions, along with their uncertainties, can be obtained only when the most relevant conformations have been extensively sampled. A particular benefit of ESMACS is the freedom to choose multiple trajectory versions to enhance predictions and provide qualitative information about and insight into the associated binding mechanisms. Most publications of MMPBSA studies use 1-trajectory approach in which conformations of the complexes, proteins and ligands are all extracted from single simulations of the complexes. The multiple trajectory versions of the approach require separate simulations for complexes, proteins and ligands, and take into account the flexibility and conformational changes of the proteins and ligands upon binding. Such multiple trajectory versions of ESMACS can significantly improve the predictions compared with those from the 1-trajectory approach when ‘induced fit’ of a ligand is a key feature of the recognition mechanism [18,19]. Relative binding free energies in the alchemical free energy domain have also attracted significant interest, particularly for drug design and drug discovery programmes. Methods for the calculation of these values include, but are not limited to, free energy perturbation (FEP)-based approaches [23,24], which have shown some potential for predicting binding affinities at the accelerated time frames needed for drug discovery, although their accuracy remains inconclusive. We have introduced a method, TIES [25] (thermodynamic integration with enhanced sampling), that makes use of ensemble techniques to ensure reproducibility, accuracy and precision in the calculation of relative binding free energies and to control the errors associated with alchemical predictions; it compares favourably with commercially offered solutions based on FEP [8]. ESMACS can be applied to highly diverse sets of ligands [2,14,16,17,19] whereas TIES is applicable to pairs of ligands of similar chemical structure. Hence ESMACS is suitable for hit-to-lead structure identification in drug discovery, while TIES has a key role in lead optimization [2,8,25]. We emphasize that both TIES and ESMACS have the advantage of being more reproducible and reliable because of the ensemble-based approaches that both protocols use [9,20]. While this certainly increases computational cost, running ensemble simulations in parallel on powerful computers reduces the wall clock time to one relevant to timescales for drug discovery and personalized medicine [18,25,26].

Both ESMACS and TIES have been employed on a variety of globular proteins, including kinase domains of different proteins, HIV proteases, peptide-MHC, bromodomains and so on [3,8,18,19,21,25–28]. To test the accuracy of computational predictions of binding affinities on membrane proteins, we have elected to use GPCRs because of their importance to the academic community and to the pharmaceutical industry. GPCRs comprise the single biggest drug target [29] with many unexploited receptors remaining to be used for drug discovery, making the calculation of accurate binding affinities an important means by which to improve the number of drugs that successfully progress from the development pipeline to the clinic. Interestingly, and despite the wealth of published experimental data that exist for this receptor superfamily, there have been relatively few studies that report the computational prediction of the binding affinity of a ligand to its target GPCR [30–34], providing us with the opportunity to compare computational calculations of binding affinities using ESMACS and TIES with published experimental results.

Proteins, generally, and GPCRs, in particular, are dynamic and function in a complex energy landscape, possessing different conformational states and interconverting between these in response to the available free energy of the system [35,36]. They can be considered to exist ‘mainly as a group of structures not too different from one another in free energy, but frequently differing considerably in energy and entropy’ [37]. The conformational changes that GPCRs undergo are elicited in response to interactions between the receptor and the ligands that bind specifically to it and to interactions between the receptor and additional proteins involved in the signalling process, including G proteins. Binding affinities and receptor conformations are inextricably intertwined. The advent of high-resolution X-ray crystal structures for GPCRs in active and inactive physiological states [38] has provided an unprecedented opportunity to examine the structural coverage of binding sites and receptor–ligand interactions [39] and affords a means by which to explore the conformational states and sub-states of these receptors, a number of which can be correlated with receptor activity. It is somewhat ironic that the experimental confirmation of the multiple active states predicted from the quantitative mathematical models of GPCRs has been provided by X-ray crystallography, a technique that emphasizes a single static macromolecule. However, the available active state GPCR X-ray crystal structures vary between crystal structures of the same GPCR and between those of different GPCRs in a manner that may be attributed either to the formation of an intermediate state which precedes the existence of a fully active state, or to the detection of one of several active structures of the GPCR [40].

We have explicitly chosen to interrogate the A_1_ and A_2A_ adenosine receptors for this work as high-resolution X-ray crystal structures of both the active and inactive forms [38] are available, substantial amounts of kinetic binding data exist and these are GPCRs with which we are familiar experimentally [41–43]. Our findings are broadly applicable to other GPCRs and to other, different cell surface receptors. The automation of the ESMACS and TIES protocols within our binding affinity calculator (BAC) [44] allows the rapid generation of binding affinities for GPCRs and other receptor protein families of interest.

## 2. Methods

In this section, we first describe the set-up of the simulations before explaining the two methodologies used to predict binding affinity values.

### 2.1. Creation of receptor models

**Figure 1.**
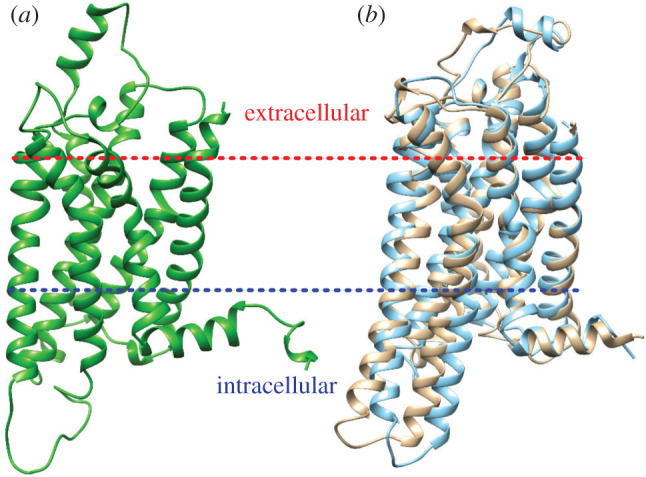
Structures of the (*a*) inactive A_1_ (PDB accession number: 5UEN) and (*b*) inactive (beige) and active (blue) A_2A_ receptors (PDB accession numbers: 5IU4 and 4UHR, respectively) in cartoon representation.

The computation of accurate binding affinities depends upon having both an accurate model of the target protein and accurately predicted poses for the ligands. GPCR structures are highly plastic, frequently adopting different conformations depending on the type of ligand to which they are bound. Three different states have been identified experimentally for the A_2A_ receptor depending on its binding partners, inactive (antagonist bound), active (also referred to as partially active, agonist bound) and fully active (in complex with a G-protein) [45,46]. The closely related A_1_ receptor is believed to explore similar states but as yet structures only exist for the inactive and fully active states [47]. The G-protein complexed (fully active) form of the receptors is extremely large and we excluded it from investigation on the grounds of computational cost. In the absence of a suitable active state structure on which to base our models, we chose to simulate A_1_ receptor agonists in the inactive state model (figure 1).

All available structures of the A_1_ and A_2A_ adenosine receptors are incomplete; all structures contain unresolved loop regions and incorporate mutations designed to facilitate crystallization. In order to obtain complete and wild-type structures for simulation, we employed the homology modelling functionality of the Molecular Operating Environment (MOE) package. The wild-type sequences of both receptors were taken from the GPCR database (gpcrdb.org) [48]. The following PDB structures were used as templates for the modelling of the different receptor states: 4UHR (CGS21680 bound) [45], 5IU4 (ZM-241,385 bound) [49], for the active and inactive forms of the A_2A_, and 5UEN [50] (co-crystallized with a ligand with no kinetic binding data) for the inactive form of the A_1_. The complete models of the two receptors, used in the study, are shown in figure 1.

GPCRs are membrane proteins. To ensure physiologically relevant simulations the models we have generated must be inserted into appropriate ligand membranes. Coordinate models of the membrane bound protein were generated within CHARMM [51] using a temporary CGENFF [52] parametrization for the ligands. A 100% DPPC lipid bilayer was generated around each receptor using the replacement method based on scripts adapted from the CHARMM-GUI membrane builder [53]. Each protein–membrane model was solvated in a tetragonal box containing TIP3P water molecules [54]. The ParmEd tool from AmberTools 16 [55] was then used to convert the systems to use the Amber FF14SB [56] forcefield for the protein and Lipid 14 [57] for the membrane (water remained parametrized using TIP3P). Histidines were assigned standard AMBER protonation states for a pH 7 environment. Final box dimensions for the A_1_ receptor were 86 × 86 × 138 Å, with 100 and 97 lipid molecules in the top and bottom layers of the membrane, respectively. Box dimensions for the inactive and active A_2A_ receptor models were 76 × 76 × 132 Å and 77 × 77 × 132 Å, respectively, with 76 lipid molecules in the top layer and 77 in the bottom layer of the membrane. All systems were examined to identify any water molecules trapped in the centre of the bilayer which were then removed. Counter ions were added to neutralize the simulation boxes, with 12 Cl^–^ and 9 Cl^–^ for the A_1_ and A_2A_ receptors, respectively.

### 2.2. Molecular dynamics simulations

MD simulations were then performed using the NAMD 2.10 package [58]. Periodic boundary conditions were applied, the Particle Mesh Ewald method [59] was applied for long range electrostatics and a Lennard-Jones cut-off of 12 Å employed. The Langevin thermostat [60] was used with a low damping coefficient of 1 ps^−1^ to keep the fluctuations between the current temperature and the target temperature to a minimum. Simulations were run at 310.15 K, the human physiological temperature, to mimic the behaviour of these receptors *in vivo*. Langevin piston control [61] was used with a damping period set to 50 fs and a time-decay period of 20 fs to maintain the pressure at 1 atm. Snapshots were saved every 1 ps for all simulations. Furthermore, all covalent hydrogen bonds were constrained using the SHAKE algorithm [62].

30 000 steps of energy minimization were performed for each system using the default conjugate gradient-coupled line search algorithm. An equilibration protocol (table 1) was followed for a total of 12.5 ns during which the first 3 ns were performed in the NVT (constant number of atoms, volume and temperature) ensemble to let the lipid molecules adjust to the volume space, and the remaining 9.5 ns were performed in the NPT (constant number of atoms, pressure and temperature) ensemble to mimic the biological experimental setting. Velocity rescaling was performed every 500 steps while applying constraints to the backbone, side-chains, and the heavy atoms of membrane lipid heads and tails. Constraints were slowly released towards the end of equilibration as described in table 1. The last 4 ns of equilibration for each system was performed in an NPT ensemble to allow sufficient time for the complexes to relax, adjust and adopt initial stable configurations in the absence of restraints. The final frame of the simulation was used in all docking and subsequent simulations.

**Table 1.** Description of the equilibration protocol and the harmonic constraints applied per step in the simulation set-up.

step	time step (fs)	ensemble	equilibration time (ns)	harmonic constraints (kcal mol^−1^ Å^−2^)				
				backbone	sidechains	lipid heads	lipid tails	ions
1	1	NVT	1	10	5	2.5	2.5	5
2	1	NVT	1	5	2.5	2.5	2.5	0
3	1	NVT	1	2.5	1	1	1	0
4	2	NPT	2	1	0.5	0.5	0.5	0
5	2	NPT	2	0.5	0.1	0.1	0.1	0
6	2	NPT	1.5	0.1	0	0	0	0
7	2	NPT	4	0	0	0	0	0

### 2.3. Ligand dataset

**Figure 2.**
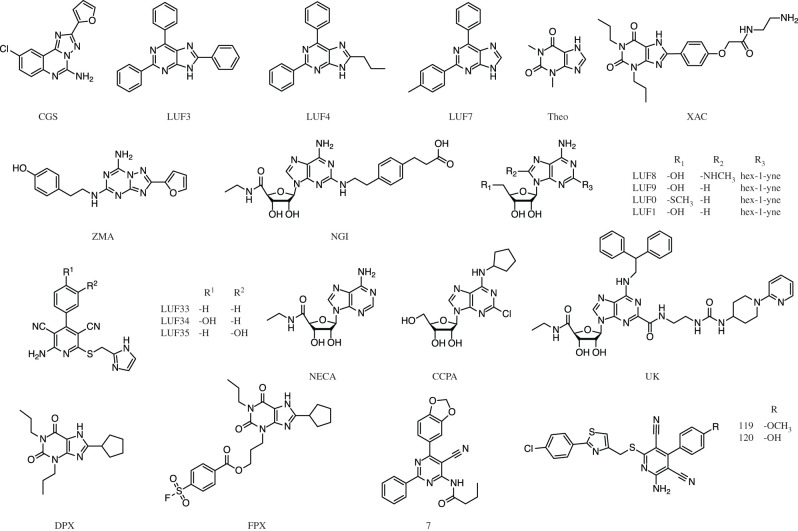
The structures of the ligands used in our study with the shortened names corresponding to table 2.

The ligands in the dataset (table 2 and figure 2) were chosen as they all had binding affinities determined using kinetic radioligand binding assays. The dataset is highly diverse comprising both agonists, antagonists and inverse agonists. In addition, eight of the ligands have experimental binding affinity values determined for both the A_1_ and A_2A_ receptor, enabling experimentally determined receptor-relative selectivity binding free energies to be calculated.

**Table 2.** Table of ligands used in this study including associated experimental binding affinity data. The PDB column contains the PDB accession number of A_2A_ receptor structures from which we extract three-dimensional ligand binding poses.

ligand name	abbreviation	ligand type	PDB	experimental binding free energies (kcal mol^−1^) [62–69]	
				A_2A_	A_1_
CGS15943	CGS	antagonist	—	−12.70 ± 0.06	−12.49 ± 0.10
LUF5834	LUF34	agonist	—	−9.77 ± 0.25	−11.53 ± 0.10
LUF5963	LUF3	antagonist	—	−8.70 ± 0.15	−10.96 ± 0.05
LUF5964	LUF4	antagonist	—	−9.28 ± 0.42	−12.59 ± 0.09
LUF5967	LUF7	antagonist	—	−8.54 ± 0.33	−12.03 ± 0.10
NECA	NECA	agonist	2YDV	−9.52 ± 0.13	−8.69 ± 0.13
theophylline	Theo	antagonist	5MZJ	−7.16 ± 0.09	−7.68 ± 0.11
XAC	XAC	antagonist	3REY	−10.11 ± 0.15	−10.86 ± 0.06
CGS21680	NGI	agonist	4UHR	−8.14 ± 0.09	—
LUF5448	LUF8	agonist	—	−8.49 ± 0.15	—
LUF5549	LUF9	agonist	—	−9.90 ± 0.14	—
LUF5550	LUF0	agonist	—	−8.84 ± 0.15	—
LUF5631	LUF1	agonist	—	−9.17 ± 0.20	—
LUF5833	LUF33	agonist	—	−9.83 ± 0.27	—
LUF5835	LUF35	agonist	—	−9.85 ± 0.26	—
UK-432,097	UK	agonist	3QAK	−10.31 ± 0.07	—
ZM-241,385	ZMA	inverse agonist	5IU4	−11.71 ± 0.09	—
LUF6057	7	agonist	—	—	−11.19 ± 0.15
CCPA	CCPA	agonist	—	—	−9.59 ± 0.10
CHEMBL3613119	119	agonist	—	—	−11.64 ± 0.10
CHEMBL3613120	120	agonist	—	—	−11.17 ± 0.22
DPCPX	DPX	inverse agonist	—	—	−12.11 ± 0.07
FSCPX	FPX	antagonist	—	—	−11.91 ± 0.14

In order to accurately compare the computational predictions of binding values to the experimental results, the experimentally determined equilibrium dissociation constants, *K*_D_, were converted into Gibbs free energies using the equation

2.1 $$\Delta G=-RT\ln K_{D}.$$

The binding free energies calculated from the experimentally determined *K*_D_ for all ligands are shown in table 2. Where the same ligand had its *K*_D_ value measured in multiple publications, the average value is shown.

### 2.4. Ligand parametrization and docking

Crystal structures of 6 ligands in our datasets are available in complex with the A_2A_ receptor (table 2). In these cases, we use those conformations in our modelling. For the remaining ligands, the structures were manually produced and optimized in IQMol [71].

All ligands were parametrized using the Antechamber component of AmberTools 16 [72,73]. For ESMACS ligands charges were obtained using the AM1BCC approach and parametrized using the general AMBER force field (GAFF) [73]. Individual ligand topologies employed in the creation of hybrid topologies used in TIES were also parametrized with GAFF but partial charges were derived by invoking the RESP algorithm following geometry optimization in Gaussian09 [74] (employing a Hartree–Fock wavefunction with a 6 − 31 + G* basis set).

For the six ligands bound to A_2A_ for which crystal binding poses were available (table 2), the experimental crystal docking poses were retained by aligning the experimental structure with the appropriate target structure. All other ligands were docked into the binding pocket of their respective receptors using the AutoDock Vina [75] plugin in the UCSF Chimera [76] package. Single binding poses were chosen on the basis of the best docking score obtained, which showed that all of the ligands (including the antagonists) bind to the orthosteric binding site. This agrees with the experimental observation from a high number of co-crystallized structures and site-directed mutagenesis binding data. We would like to point out that in cases where a ligand may target multiple binding sites, or target an allosteric pocket, the metadynamics protocol [5,6] may be useful. In the remainder of this work, we refer to a complete parametrized model containing protein, equilibrated membrane and docked ligand as a ‘starting structure’.

### 2.5. Binding free energy protocols

Here, we use two computational techniques to gain information about ligand binding strengths: ESMACS, which ranks absolute binding free energies (ΔG) directly, and TIES, which computes differences in Gibbs free energies between two related systems (ΔΔG). The set-up of the simulations for each protocol is substantially different and is described in detail below. Simulation execution and analysis for both protocols were automated via our BAC [44] workflow tool.

### 2.6. ESMACS

ESMACS protocols are designed to provide converged binding free energies calculated using the MMPBSA methodology from ensembles of relatively short duration simulations for diverse ligand datasets. They include a range of methodologies to compute the entropic contribution to binding usually neglected in standard MMPBSA approaches and may also account for ligand and receptor flexibility using multiple trajectories, including not only that of the complex but those of the unbound ligand and apo protein.

The binding free energy associated with the binding of a ligand to its target protein is calculated as follows:

2.2 $$\Delta G=\langle G^{\mathrm{Complex}}\rangle -\langle G^{\mathrm{receptor}}\rangle -\langle G^{\mathrm{ligand}}\rangle \;,$$

where $\left\langle G^{\mathrm{Complex}}\right\rangle$, $\left\langle G^{\mathrm{receptor}}\right\rangle$ and $\left\langle G^{\mathrm{ligand}}\right\rangle$ are the average values of the free energy contribution from complex, receptor (protein) and ligand respectively. Traditionally, in MMPBSA calculations, sampling is conducted using simulations of the complex alone.

The estimate of the component free energy provided by ESMACS can be decomposed as follows:

2.3 $$\begin{array}{rl} G_{\mathrm{ESMACS}}^{i} & =G_{\mathrm{MMPBSA}}^{i}-TS_{\mathrm{conf}}^{i}\\ & =E_{\mathrm{MM}}^{i}+G_{\mathrm{solvent}}^{i}-TS_{\mathrm{conf}}\\ & =E_{\mathrm{int}}^{i}+E_{\mathrm{vdW}}^{i}+E_{\mathrm{ele}}^{i}+G_{\mathrm{PB}}^{i}+G_{\mathrm{SA}}^{i}-TS_{\mathrm{conf}}, \end{array}\}$$

where $E_{\mathrm{MM}}^{i}$ is the molecular mechanical energy contribution of the species *i*, in a *complex*, free *receptor* or unbound *ligand* in the gas phase. *S*_conf_ is the configurational entropy. This comprises internal bonded energies ($E_{\mathrm{int}}^{i}$), van der Waals ($E_{\mathrm{vdW}}^{i}$), and electrostatic interactions ($E_{\mathrm{ele}}^{i}$). $G_{\mathrm{solvent}}^{i}$ is the solvent free energy term estimated from the sum of the Poisson–Boltzmann ($G_{\mathrm{PB}}^{i}$) and the non-polar solvation free energy terms ($G_{\mathrm{SA}}^{i}$). $G_{\mathrm{SA}}^{i}$ is calculated from the solvent accessibility surface area (SASA) using

2.4 $$G_{\mathrm{SA}}^{i}=\gamma \times \text{SASA}+b,$$

where *γ* is the surface tension, and *b* the offset (we use the default values of 0.00542 kcal mol^−1^ Å^−2^ and 0.92 kcal mol^−1^ respectively [77]). The entropic term is introduced as the product of the temperature (*T*) and configurational entropy. The most common method of computing the configurational entropy is through normal mode analysis [78,79]. However, converging these calculations is computationally demanding for large systems [18] (potentially using as many computational resources as the original molecular dynamics calculations). This motivated the creation of an alternative solution: the weighted solvent accessible surface area (WSAS) model [19,80]. This method was parametrized to reproduce normal mode analysis results from computationally cheap atomistic surface area calculations. In this approach the solvent accessible surface area (SAS) and buried surface area (BSAS) are weighted according to atom type and the sum of the contributions of each atom is used to estimate *S*_conf_ as per the following relationship:

2.5 $$S_{\mathrm{conf}}^{\mathrm{WSAS}}=\overset{N}{\underset{i=1}{\sum }}w_{i}(\text{SA}{\text{S}}_{i}-k\text{BSA}{\text{S}}_{i}),$$

where *w_i_* is the atom-type specific weighting of the atom *i* and *k* is a scaling parameter of BSAS. BSAS for atom *i* is computed using:

2.6 $$\text{BSA}{\text{S}}_{i}=4\pi {\left(r_{i}+r_{\mathrm{prob}}\right)}^{2}-\text{SA}{\text{S}}_{i},$$

where *r_i_* is the radius of the atom *i*, and *r*_prob_ the probe radius of a water molecule. Here, we compute the surface areas using the Lee and Richards algorithm [81] as implemented in freesasa [80].

The starting structure generated for each protein–ligand was used to initiate ESMACS runs using a protocol modified from that used for previous work on globular proteins [3,16,18]. In each run 25 replica simulations were executed varying only by initial velocities, which were randomly drawn from the Maxwell–Boltzmann distribution. Each run was initialized with weak harmonic constraints (of up to 3 kcal mol^−1^ Å^−2^) applied to the receptors' backbone and ligand's heavy atoms, which were slowly released during 0.5 ns of equilibration. Following this, production simulations were instigated. The same NAMD settings were used in production simulations as for the NPT steps of the membrane equilibration protocol.

Between 30 and 45 ns were required for convergence for models containing docked ligands, with slightly more rapid convergence for the ligands using poses copied from crystal structures (up to 28 ns). This is around 10 times longer than the protocol used in previous ESMACS studies and is due to the complex nature of GPCRs. The binding free energies predicted from ESMACS were based on 50 uniformly distributed frames across the last 10 ns of each of the 25 replicas and then averaged.

### 2.7. TIES

TIES is based on thermodynamic integration (TI), a well-established example of so-called alchemical binding free energy methods [82–85]. Alchemical free energy calculations employ unphysical (alchemical) intermediates to calculate changes in free energies between two physically real systems. It is common in these methods to refer to a variable, *λ*, which describes the path taken to transform one ligand into another. The parameter varies between 0 and 1, with 0 representing the initial ligand, L1, and 1 the final ligand, L2. The potential between these endpoints is given by

2.7 $$V(\lambda ,x)=(1-\lambda )V_{1}(\lambda ,x)+\lambda V_{2}(\lambda ,x)\;,$$

where *V*_1_ and *V*_2_ are the potential energies of L1 and L2, respectively, and *x* represents the coordinates of the system. The derivative of the hybrid potential energy with respect to *λ*, ∂V(λ,x)/∂λ, is used to compute the free energy difference using

2.8 $$\Delta G_{TI}=\int_{0}^{1}{\left\langle \frac{\partial V(\lambda ,x)}{\partial \lambda }\right\rangle }_{\lambda }\;\text{d}\lambda ,$$

where ${\left\langle \ldots \right\rangle }_{\lambda }$ denotes the ensemble average at the chosen *λ*. In practice, the integral is calculated numerically, with MD sampling used in the computation of the ensemble averages at a set of discrete points (the so-called λ-windows). In TIES, multiple replica MD simulations are performed at each λ-window.

We employ a thermodynamic cycle approach to calculate relative free energy difference (ΔΔG_TIES_) between two ligands:

2.9 $$\Delta \Delta G_{\mathrm{TIES}}=\Delta G_{1}-\Delta G_{2}=\Delta G_{TI}^{\mathrm{aqueous}}-\Delta G_{TI}^{\mathrm{bound}},$$

where ΔG_1_ and ΔG_2_ are the binding energies for L1 and L2, respectively. $\Delta G_{TI}^{\mathrm{aqueous}}$ and $\Delta G_{TI}^{\mathrm{bound}}$ are the free-energy components resulting from the alchemical transformation of L1 to L2 in the unbound and bound states.

As described in previous work [25], treating the integrals in equation (2.9) through the lens of stochastic calculus provides a robust method to estimate uncertainties. The ensemble average of the potential derivative is calculated as the average of its values from all replica simulations in our ensemble simulation, where the individual value for each replica is taken to be the average potential derivative over the whole simulation length. The error associated with each λ-window is computed as a bootstrapped standard error of the mean of the *λ* derivatives from all sampled replicas using

2.10 $$\sigma_{x=\mathrm{aqueous},\mathrm{bound}}^{2}=\underset{\lambda }{\sum }\sigma_{\lambda }^{2}{\left(\Delta \lambda \right)}^{2},$$

where $\sigma_{\lambda }^{2}$ is the variance associated with the relevant λ-window in the aqueous or bound calculation, as appropriate. This error is the convolution of the individual errors for each *λ* window. The overall error, *σ*, then is computed using

2.11 $$\sigma^{2}=\sigma_{\mathrm{aqueous}}^{2}+\sigma_{\mathrm{bound}}^{2}.$$

The domain of validity of TIES targets resides in determining differences in binding free energies between closely chemically related (for example congeneric) ligands between which there are no charge differences. A list of ligand pairs which meet these criteria in our experimental dataset is provided in table 3.

**Table 3.** The ligand pairs (L1 and L2) for which TIES calculations were performed in this study and their associated experimentally determined relative binding affinities (ΔΔG).

transformation		ΔΔG_Experiment_ (kcal mol^−1^)	
L1	L2	A_2A_	A_1_
LUF3	LUF4	−0.58 ± 0.45	−1.63 ± 0.10
LUF3	LUF7	0.16 ± 0.36	−1.07 ± 0.11
LUF4	LUF7	0.74 ± 0.53	0.56 ± 0.13
Theo	XAC	−2.95 ± 0.17	−3.18 ± 0.13
LUF8	LUF1	−0.68 ± 0.25	—
LUF34	LUF35	−0.08 ± 0.36	—
LUF33	LUF34	0.06 ± 0.37	—
LUF33	LUF35	−0.02 ± 0.37	—
NECA	CCPA	—	−0.90 ± 0.16
119	120	—	0.47 ± 0.24
XAC	DPX	—	−1.25 ± 0.09

A hybrid ligand topology must be created based on the chemically common region, a disappearing domain comprising the atoms only present in L1 and an appearing domain containing atoms unique to L2. We generated initial common region for each pair of ligands using FESetup [86] and used these to align the two ligands. Atoms were removed from the common region if their charge differed by more than 0.1*e*. The partial atomic charges for the hybrid ligand were obtained from the RESP derived partial atomic charges on the individual ligands such that the common atoms had identical charges, taken to be the average of their charges in the individual ligands. The charges on disappearing and appearing parts were then adapted by reparametrizing the ligands after constraining the charges on the common atoms to their new values.

We deployed the same basic TIES protocol set out by Bhati *et al.* [25], using 5 replica simulations in each of 13 non-interacting λ-windows, placed at: 0, 0.05, 0.1, 0.2, 0.3, 0.4, 0.5, 0.6, 0.7, 0.8, 0.9, 0.95 and 1.0. We employed a soft-core potential [87,88] for the van der Waals interactions to prevent divergent potential energies, which may arise when there are sudden appearances or disappearances of atoms close to the endpoints of the alchemical transformations. The electrostatic interactions of the disappearing atoms were linearly decoupled from the simulations between *λ* values of 0 and 0.55 and then turned off, while those of the appearing atoms were linearly coupled from *λ* values of 0.45 to 1 and then fully activated.

The hybrid ligands were docked into the target structures using the same approaches employed for the single ligands within the ESMACS calculations. Each replica simulation was instigated from the starting structure and varied only by the initially randomized velocities [25]. To ensure accurate and precise values of ΔΔG_TIES_, a three-step equilibration process lasting 2 ns was performed at each *λ* window. The integration time steps over the three equilibration periods were 0.5, 1 and 2 fs, respectively. The electrostatic interaction energies were computed at every step to ensure integrator stability. As the TIES protocol only computes the difference in binding free energy between two similar ligands, 4 ns production runs were performed, as done for other systems within the TIES protocol [2,25]. All molecular dynamics simulations were performed using NAMD with the same thermostat and barostat settings as applied in ESMACS and the membrane equilibration protocol.

## 3. Results

In this section, we present binding affinity predictions from both ESMACS and TIES.

### 3.1. ESMACS

**Figure 3.**
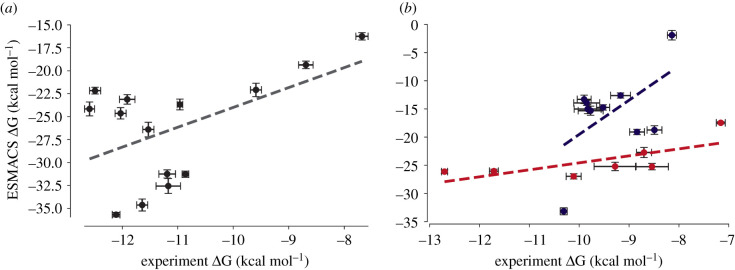
Summary of results obtained using ESMACS for ligands of the (*a*) A_1_ and (*b*) A_2A_ receptors. The grey dashed line in (*a*) is the linear correlation line for all the ligands. For the A_2A_ receptor (*b*), agonists and antagonists are coloured blue and red, respectively. The two dashed lines in (*b*) show the linear correlation for agonists and antagonists, also coloured blue and red, respectively.

Predictions of binding free energy (ΔG), using the ESMACS protocol, were carried out on 14 and 17 ligands of the A_1_ and A_2A­_ receptors, respectively (structures and experimentally determined data shown in figure 2 and table 2). The results of the predicted ΔG compared to experimentally determined data are shown in figure 3. As there is an approximately 1 kcal mol^−1^ range of experimentally determined ΔG values available from different publications, it is likely that the associated errors are underreported. For the 14 A_1_ receptor ligands, the correlation obtained is reasonable (*R*_P_ = 0.53). The overall correlation for the A_2A_ receptor ligands is slightly weaker (*R*_P_ = 0.43). As ‘active’ and ‘inactive’ starting structures were used to calculate ΔG values for agonists and antagonists (and inverse agonists), respectively, use of two separate correlation lines is more appropriate for the A_2A_ receptor. This results in stronger correlations of *R*_P_ = 0.73 for the antagonists (and the inverse agonist) and *R*_P_ = 0.55 for the agonists. If one includes the configurational entropy in the ESMACS ΔG binding free energies predictions, the A_1_ and A_2A_ correlations become weaker, with the exception of the A_2A_ agonists which retains the same strength in correlation (*R*_P_ = 0.55). Overall ESMACS performs similarly well for structurally diverse A_1_ ligands, A_2A_ agonists and A_2A_ antagonists and better without inclusion of the configurational entropy.

### 3.2. TIES

**Figure 4.**
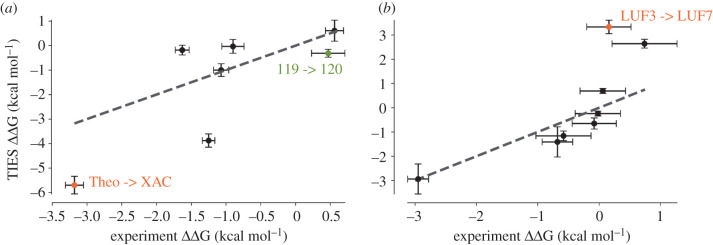
Plot of computed relative binding free energies using TIES (TIES ΔΔG) against experimentally determined relative binding free energies for ligand pairs of the (*a*) A_1_ and (*b*) A_2A_ receptor. All reported ligand transformations are listed in table 3. Values were classified as outliers when their Cook's distance [89] was larger than 4/*n*, where *n* represents the total number of points used in the regression. These outliers are labelled and plotted in orange. The dotted line plots *y* = *x*, representing correct prediction values.

Relative binding free energies (ΔΔG) were calculated using TIES for 7 and 8 pairs of ligands for the A_1_ and A_2A_ receptor, respectively (table 3). These 15 ligand pairs were the only ligands structurally similar enough in the data (table 2) that we were able to calculate hybrid topologies. To our knowledge, this is the first published use of an alchemical binding free energy prediction method on ligands of the A_1_ receptor. The results of these relative binding free energy calculations against experimentally determined data are plotted in figure 4. As TIES aims to calculate relative binding free energies, the correlation line was set as *y* = *x*. The overall mean absolute error was calculated as 1.2 and 0.98 kcal mol^−1^ for all ligands of the A_1_ and A_2A_ receptors, respectively. This is similar to values reported previously using TIES on much simpler, non-membrane proteins [25]. All but one of the predicted ΔΔG values are directionally correct.

Furthermore, only one transformation pair's predicted ΔΔG values, from each receptor subtype, was classified as an outlier from its true value when its Cook's distance [89] was greater than 4/*n* (*n* being the number of points used in the regression line). These pairs are highlighted in orange in figure 4. One of the outliers, the Theo -> XAC ligand pair in the A_1_ receptor, is the largest alchemical transformation performed in our TIES calculations, as these ligands are the most structurally divergent among all the ligand pairs and therefore the least reliable calculation. Excluding these outliers, the mean absolute error improves to 0.98 and 0.66 kcal mol^−1^ for remaining A_1_ and A_2A_ ligand pairs, respectively. This is similar to the weighted mean absolute errors achieved using alchemical free energy calculations on two ligand series of the A_2A_ receptor [31].

## 4. Conclusion

Using the TIES and ESMACS protocols, we have computed the binding free energies of a series of ligands at the A_1_ and A_2A_ adenosine receptors, two GPCRs for which substantial quantities of structural and functional data exist. Our rankings for binding free energies determined by both ESMACS and TIES are in line with previous experiments, confirming our ability to use these protocols on GPCRs, which are much larger protein targets than used previously with these protocols. ESMACS predicts values that correlate well with experimentally determined binding free energy values for structurally diverse sets of ligands, confirming its value for the hit-to-lead phase of drug discovery. TIES is again found to be a powerful protocol for the accurate calculation of relative binding free energies between structurally similar ligands, and is superior to methods that do not use equivalent ensemble-based sampling techniques, such as FEP+ [8]. TIES is thus of considerable value in lead optimization; here, we demonstrated this in a relatively extreme case involving a complex protein class with large alchemical transformations and correspondingly small common substructures. Although drug design has numerous constraints, we conclude that this methodology provides a useful tool with which to inform structure-based drug discovery workflows for the development of novel GPCR therapeutics.

## Data accessibility

This article has no additional data.
